# Intensive care unit admission from the emergency department in the setting of National Emergency Access Targets

**DOI:** 10.1016/j.ccrj.2023.05.005

**Published:** 2023-06-30

**Authors:** Jack D'Arcy, Suzanne Doherty, Luke Fletcher, Ary Serpa Neto, Daryl Jones

**Affiliations:** a Austin Health, Melbourne, Australia; b Data Analytics Research and Evaluation (DARE) Centre, University of Melbourne, Melbourne, VIC, Australia; c Department of Critical Care, The University of Melbourne, Melbourne, VIC, Australia; d Australian and New Zealand Intensive Care Research Centre, School of Public Health and Preventive Medicine, Monash University, Melbourne, VIC, Australia; e Department of Critical Care Medicine, Hospital Israelita Albert Einstein, São Paulo, Brazil; f School of Public Health and Preventive Medicine, Monash University, Melbourne, Victoria, Australia; g The University of Melbourne, Melbourne, Victoria, Australia

**Keywords:** NEAT, Length of stay, Intensive care unit (ICU), Emergency department (ED), Admission, Access block

## Abstract

**Purpose:**

Since the introduction of National Emergency Access Targets (NEATs) in 2012 there has been little research examining patients admitted to the intensive care unit (ICU).

We assessed differences in baseline characteristics and outcomes of patients admitted from the Emergency Department (ED) to the ICU within 4 hours compared with patients who were not.

**Methods:**

This retrospective observational study included all adults (≥18 years old) admitted to the ICU from the ED of Austin Hospital, Melbourne, Australia, between 1 January 2017 and 31st December 2019 inclusive.

**Results:**

1544 patients were admitted from the ED to the ICU and 65% had an ED length of stay (EDLOS) > 4 hour. Such patients were more likely to be older, female, with less urgent triage category scores and lower illness severity. Sepsis and respiratory admission diagnoses, and winter presentations were significantly more prevalent in this group.

After adjustment for confounders, patients with an EDLOS > 4 hours had lower hospital mortality; 8% v 21% (p = 0.029; OR, 1.62), shorter ICU length of stay 2.2 v 2.4 days (p = 0.043), but a longer hospital length of stay 6.2 v 6.8 days (p = < 0.001).

**Conclusion:**

Almost two thirds of patients breached the NEAT of 4 hours. These patients were more likely to be older, female, admitted in winter with sepsis and respiratory diagnoses, and have lower illness severity and less urgent triage categories. NEAT breach was associated with reduced hospital mortality but an increased hospital length of stay.

## 1 Introduction

Prolonged emergency department length of stay (EDLOS) has been associated with adverse outcomes including delayed treatment, prolonged hospitalisation, and increased mortality in some studies.[1], [2], [3], [4] This may have implications for critically ill patients who require intensive care unit (ICU) admission.

However, research evaluating prolonged EDLOS is conflicting, with many studies reporting increased mortality, prolonged hospital admission, and persistent organ dysfunction,[5], [6], [7], [8], [9], [10], [11], [12] while others report no difference in mortality.[13], [14], [15], [16]

An important consideration in analysing access block and its adverse sequelae in Australia has been the introduction of the National Emergency Access Target (NEAT) policy in 2012, which stated that 90% of patients presenting to the ED should be admitted, transferred, or discharged within 4 h of ED presentation, in-line with policy established earlier in the UK.[17], [18], [19]

This intervention appears to have reduced EDLOS and improved ED function.^18^ The effect on in-hospital mortality is controversial and where association with improved mortality is demonstrated, it appears to be jurisdiction dependent^18^ and without a clear understanding of what level of NEAT compliance is most beneficial.^20^

The impact of NEAT on patients requiring ICU admission has been less extensively investigated. A retrospective analysis found that elderly patients with complex medical conditions, patients presenting after-hours and on weekends, and those with time-sensitive cardiorespiratory presentations all have improved mortality with NEAT introduction.^21^ In addition, implementation of a code response to expedite ICU admission reduced EDLOS and in-hospital mortality.^22^

Given the sparsity of data and conflicting outcomes, we evaluated the epidemiology of patients admitted directly to ICU from the ED in the setting of NEAT and evaluated key outcome measures of patients admitted within 4 h s, compared with those that were not.

## 2 Methods

### 2.1 Ethics approval

This study was approved by the Human Research Ethics Committee (Audit/21/Austin/40). Due to the observational and retrospective nature of the study, the need for informed written consent from participants was waived. The STROBE recommendations were followed for the reporting of observational studies.^23^

### 2.2 Study design

This was a retrospective observational study that included all adult patients (≥18 years old) admitted to the ICU from the ED of Austin Hospital, Melbourne, Australia, between 1 January 2017 and 31 December 2019 inclusive. This period was chosen to avoid the confounding effects of the COVID-19 pandemic.

We excluded patients admitted to ICU via the operating theatre, the interventional radiology suite and cardiac catheter laboratory, the general wards, the emergency short stay unit, and patients who died in the ED.

EDLOS was measured from hospital informatic systems that record the time of hospital presentation and discharge from the ED. These data are entered by administrative staff as part of routine care.

### 2.3 Setting

Austin Hospital is a major teaching hospital affiliated with the University of Melbourne and admits approximately 26,000 multiday admissions each year. Approximately 88,000 patients attend the ED annually with a hospital admission rate of approximately 40%. It is the state referral centre for liver transplantation, aortic surgery, spinal cord injuries, and ventilatory weaning. The ICU has 23 beds and admits approximately 2200 admissions per year. The ICU is staffed 24/7 by registrars (fellows), and a consultant (attending) is present for at least 10 h per day on weekdays.

### 2.4 Data source

Data were extracted from existing databases including the hospital electronic medical record, and local data submitted to the Australian and New Zealand Intensive Care Society Adult Patient Database (ANZICS APD).^24^

### 2.5 Details of data collected

Baseline demographic data collected included age, gender, type of admission (medical, elective surgical, emergency surgical), and admission category. Data related to the ED admission included the day, time, and season of admission. We also recorded the Triage Category as classified by the Australian Triage Scale at presentation.^25^

We collected data needed to calculate the ICU illness severity scores termed the Acute Physiology And Chronic Health Evaluation II (APACHE II)^26^ and Australian and New Zealand Risk of Death s (ANZROD).^27^ Complete case-analyses was performed.

### 2.6 Objectives

The aim of the study was to evaluate the epidemiology of patients admitted directly from the ED to the ICU in the era of the NEAT. The primary objective was to evaluate the proportion of patients admitted to ICU in < 4 h. The secondary objective was to identify differences in baseline characteristics and key outcomes of patients who are admitted to ICU within 4 h compared to patients who are not.

### 2.7 Outcome measures

Outcome measures included the differences in hours of mechanical ventilation, ICU and hospital length of stay and ICU, in-hospital, and 30-day hospital mortality for patients admitted >4 h compared with patients admitted within 4 h of arrival to the ED.

### 2.8 Details of statistical analysis

Continuous variables are presented as median (quartile 25th - quartile 75th) and compared using Wilcoxon rank-sum test. Categorical variables are presented as absolute numbers and percentages and compared with Fisher exact test.

Comparison of continuous variables between the groups was performed using median regression with an interior point algorithm and reported as median difference (MD) and 95% confidence interval (CI). Binary variables were compared using logistic regression and reported as odds ratio (OR) and 95%CI. Time-to-event variables were reported in Kaplan–Meier curves, compared with Cox proportional hazard models and reported as hazard ratio (HR) and 95%CI.

Adjusted analyses were further performed using the models above and considering the following clinical variables as confounders: age, ANZROD, triage category, Charlson comorbidity index, type of admission, and use of mechanical ventilation during ICU stay. Confounders were selected based on clinical relevance and evidence.

To further understand the nonlinear impact of ED LOS and hospital mortality, additional models were performed. First, a model considering ED LOS as a linear predictor was performed. Then, ED LOS was treated with natural cubic splines and restricted cubic splines. Results are presented in marginal effect plots. In addition, a LOESS curve was performed.

Statistical significance was considered when a *p* < 0.05 was found. Analysis was performed using R v.4.0.3.

## 3 Results

### 3.1 Details of the overall cohort

Between 1 January 2017 and 31 December 2019, 1544 patients were admitted directly from the ED to the ICU. Baseline characteristics for patients included in the study are outlined in Table 1. The median age was 62 years and 56% were male. The median Charlson Comorbidity Index was 4, and 49% had a triage category of 2 (ATS). The median APACHE II score was 16 and ANZROD was 4.4%.

**Table 1:** Baseline characteristics of the included patients.

	Overall (*n* = 1544)	ED LOS < 4 h (*n* = 548)	ED LOS ≥ 4 h (*n* = 996)	*p* value
Age, years	62 (46–74)	60 (44–72)	63 (47–75)	0.004
Male gender - no. (%)	860 (55.7)	326 (59.5)	534 (53.6)	0.028
APACHE II	16 (11–22)	19 (12–25)	15 (10–20)	<0.001
ANZ risk of death, %	4.4 (1.3–15.8)	7.7 (1.4–35.7)	3.7 (1.2–10.3)	<0.001
Triage category - no. (%)				<0.001
1	321 (20.8)	233 (42.5)	88 (8.8)	
2	757 (49.0)	266 (48.5)	491 (49.3)	
3	381 (24.7)	39 (7.1)	342 (34.3)	
4	84 (5.4)	10 (1.8)	74 (7.4)	
5	1 (0.1)	0 (0.0)	1 (0.1)	
Type of admission - no. (%)				<0.001
Medical	1208/1421 (85.0)	390/506 (77.1)	818/915 (89.4)	
Elective surgery	5/1421 (0.4)	1/506 (0.2)	4/915 (0.4)	
Emergency surgery	208/1421 (14.6)	115/506 (22.7)	93/915 (10.2)	
ED arrival shift - no. (%)				0.474
AM (0800–1800)	572 (37.0)	196 (35.8)	376 (37.8)	
PM (1800-0800)	972 (63.0)	352 (64.2)	620 (62.2)	
ED arrival day - no. (%)				0.725
Weekday	1097 (71.0)	386 (70.4)	711 (71.4)	
Weekend	447 (29.0)	162 (29.6)	285 (28.6)	
ED arrival season - no. (%)				0.015
Autumn	341 (22.1)	124 (22.6)	217 (21.8)	
Spring	396 (25.6)	142 (25.9)	254 (25.5)	
Summer	393 (25.5)	159 (29.0)	234 (23.5)	
Winter	414 (26.8)	123 (22.4)	291 (29.2)	
Admission category - no. (%)				<0.001
Cardiovascular	212/1421 (14.9)	111/506 (21.9)	101/915 (11.0)	
Gastrointestinal	114/1421 (8.0)	32/506 (6.3)	82/915 (9.0)	
Haematological	10/1421 (0.7)	1/506 (0.2)	9/915 (1.0)	
Metabolic	238/1421 (16.7)	98/506 (19.4)	140/915 (15.3)	
Musculoskeletal	13/1421 (0.9)	6/506 (1.2)	7/915 (0.8)	
Musculoskeletal/Skin	1/1421 (0.1)	0/506 (0.0)	1/915 (0.1)	
Neurological	173/1421 (12.2)	113/506 (22.3)	60/915 (6.6)	
Renal/Genitourinary	58/1421 (4.1)	8/506 (1.6)	50/915 (5.5)	
Respiratory	291/1421 (20.5)	75/506 (14.8)	216/915 (23.6)	
Sepsis	262/1421 (18.4)	42/506 (8.3)	220/915 (24.0)	
Trauma	49/1421 (3.4)	20/506 (4.0)	29/915 (3.2)	
Total Charlson comorbidity index	4 (2–6)	4 (2–6)	4 (2–6)	0.008
Acute myocardial infarction	66 (4.3)	36 (6.6)	30 (3.0)	0.001
Chronic heart failure	207 (13.4)	57 (10.4)	150 (15.1)	0.010
Peripheral vascular disease	54 (3.5)	25 (4.6)	29 (2.9)	0.111
Cerebrovascular disease	140 (9.1)	108 (19.7)	32 (3.2)	<0.001
Dementia	9 (0.6)	2 (0.4)	7 (0.7)	0.505
Chronic obstructive pulmonary disease	151 (9.8)	41 (7.5)	110 (11.0)	0.025
Rheumatological disease	20 (1.3)	4 (0.7)	16 (1.6)	0.165
Diabetes without complication	89 (5.8)	35 (6.4)	54 (5.4)	0.427
Diabetes with complication	292 (18.9)	97 (17.7)	195 (19.6)	0.378
Mild liver disease	117 (7.6)	28 (5.1)	89 (8.9)	0.007
Severe liver disease	44 (2.8)	16 (2.9)	28 (2.8)	0.875
Cancer	49 (3.2)	10 (1.8)	39 (3.9)	0.032
Metastatic cancer	47 (3.0)	8 (1.5)	39 (3.9)	0.008
ED length of stay, minutes	309 (169–445)	65 (122–203)	393 (315–535)	<0.001

The most common admission diagnoses were sepsis, respiratory illness, and trauma. In relation to bed card, 85% of patients were admitted under a medical team and 14.6% of patients required emergency surgery.

The median EDLOS was 309 min. Approximately, two thirds of patients present to ED after 6 pm and 29% present to ED on a weekend.

### 3.2 Differences in baseline characteristics according ED length of stay

Overall, 65% of the patients had an ED LOS of >4 h. The median length of stay in the ED for patients admitted >4 h after presentation was 393 min and for patients admitted <4 h was 165 min.

Patients with an EDLOS >4 h were older and had a greater proportion of females compared to patients with an EDLOS <4 h.

This group also had significantly lower proportions of triage category 1 and a higher proportion of category 3 and category 4 patients, suggesting a lower acuity of illness at ED presentation.

Sepsis and respiratory admission diagnoses were significantly more prevalent in patients with an EDLOS >4 h, while cardiovascular and neurological presentations were more prevalent for patients admitted to ICU <4 h (Table 1).

A greater proportion of patients with an EDLOS of >4 h presented in winter whilst a greater proportion of patients admitted to ICU within 4 h presented in summer (Table 1).

There was no significant difference in ED arrival day, ED arrival shift, Charlson comorbidity index, or vital signs on the first day of ICU admission based on EDLOS (Table 1)

### 3.3 Differences in patient outcomes according to ED length of stay

Patients with an EDLOS >4 h also had lower illness severity scores. Thus, the APACHE II score was 15 v 19 and risk of death scores; ANZROD 3.7 v 7.7. This group also had higher pH and GCS, lower lactate, and reduced frequency of mechanical ventilator use in ICU for the first day (Table 2).

**Table 2:** ICU Patient Characteristics: 1st 24 h.

	Overall (*n* = 1544)	ED LOS < 4 h (*n* = 548)	ED LOS ≥ 4 h (*n* = 996)	*p* value
Use of mechanical ventilation - no. (%)				
First day of ICU admission	577/1415 (40.8)	331/503 (65.8)	246/912 (27.0)	<0.001
During ICU stay	600 (38.9)	333 (60.8)	267 (26.8)	<0.001
Vital signs in the first day of ICU admission				
Glasgow coma scale	15 (13–15)	14 (3–15)	15 (14–15)	<0.001
Highest temperature, ºC	37.0 (36.5–37.5)	37.0 (36.5–37.5)	37.0 (36.5–37.5)	0.960
Highest heart rate, bpm	100 (88–120)	100 (85–120)	103 (90–120)	0.170
Lowest mean arterial pressure, mmHg	65 (58–73)	66 (59–73)	65 (58–73)	0.394
Pathology in the first day of ICU admission				
pH	7.38 (7.30–7.44)	7.36 (7.28–7.42)	7.40 (7.32–7.45)	<0.001
PaO_2_/FiO_2_, mmHg	305 (200–390)	310 (199–393)	305 (203–390)	0.697
Highest creatinine, μmol/L	101 (71–167)	99 (71–158)	102 (70–169)	0.903
Lactate, mmol/L	2.4 (1.7–4.0)	2.8 (1.9–4.4)	2.3 (1.7–3.4)	<0.001

The median duration of mechanical ventilation was 31 h. There was no difference in the hours of mechanical ventilation between the two groups of patients (Table 3).

**Table 3:** Clinical outcomes.

	Overall (*n* = 1544)	ED LOS < 4 h (*n* = 548)	ED LOS ≥ 4 h (*n* = 996)	Unadjusted Analysis		Adjusted Analysis^a^	
				Effect Estimate (95% CI)	*p* value	Effect Estimate (95% CI)	*p* value
IMV during ICU stay	600 (38.9)	333 (60.8)	267 (26.8)	OR, 4.23 (3.39–5.28)	<0.001	OR, 2.35 (1.77–3.13)	<0.001
Hour of ventilation	31.0 (12.8–78.2)	28.0 (13.0–73.0)	34.0 (12.0–86.5)	MD, −6.00 (−17.05 to 5.06)	0.287	MD, −8.78 (−20.41 to 2.84)	0.139
ICU mortality - no. (%)	153 (9.9)	100 (18.2)	53 (5.3)	OR, 3.97 (2.81–5.68)	<0.001	OR, 1.57 (0.90–2.75)	0.115
Hospital mortality - no. (%)	189 (12.2)	114 (20.8)	75 (7.5)	OR, 3.22 (2.36–4.42)	<0.001	OR, 1.60 (0.98–2.61)	0.059
30-day hospital mortality - no. (%)	238 (15.4)	127 (23.2)	111 (11.1)	HR, 2.24 (1.73–2.89)	<0.001	HR, 1.06 (0.75–1.49)	0.744
ICU length of stay, days	2.2 (1.1–4.1)	2.4 (1.1–5.0)	2.2 (1.2–3.8)	MD, 0.23 (−0.12 to 0.58)	0.195	MD, −0.24 (−0.58 to 0.09)	0.150
In survivors	2.2 (1.2–4.0)	2.4 (1.1–5.0)	2.2 (1.1–3.7)	MD, 0.25 (−0.10 to 0.60)	0.160	MD, −0.24 (−0.56 to 0.09)	0.157
In non-survivors	2.1 (0.8–4.9)	2.3 (0.9–4.9)	1.9 (0.7–4.9)	MD, 0.40 (−1.11 to 1.90)	0.606	MD, 0.39 (−1.47 to 2.24)	0.683
Hospital length of stay, days	6.6 (3.2–13.4)	6.2 (2.5–13.0)	6.8 (3.7–13.4)	MD, −0.60 (−1.48 to 0.27)	0.175	MD, −1.37 (−2.25 to −0.49)	0.002
In survivors	7.0 (3.7–14.2)	7.2 (3.0–14.9)	6.9 (3.8–13.6)	MD, 0.40 (−0.67 to 1.47)	0.461	MD, −0.83 (−1.74 to 0.08)	0.074
In non-survivors	3.1 (0.9–7.5)	2.9 (1.1–6.1)	4.0 (0.9–9.8)	MD, −1.08 (−3.05 to 0.88)	0.281	MD, −0.80 (−3.67 to 2.07)	0.586

Data are median (quartile 25 - quartile 75) or N/total (%).

Abbreviation: ICU is intensive care unit, MD is median difference, OR is odds ratio and HR is hazard ratio; IMV is invasive mechanical ventilation.

a Adjusted for age, ANZ risk of death (after log transformation), triage category, total Charlson comorbidity index, type of admission and use of mechanical ventilation during ICU stay.

Regarding ICU length of stay, the median was 2.2 days, and there was no difference between the two patient groups. Patients admitted more than 4 h after presenting to ED had a longer hospital length of stay after adjustment for confounding factors (Table 3).

### 3.4 Associations between ED length of stay and mortality

Overall ICU mortality was approximately 10%, hospital mortality was 12%, and 30-day in-hospital mortality was 15%, respectively. Unadjusted mortality at all time points was higher among patients admitted in < 4h. Thus, ICU, in-hospital and 30-day mortality was higher in those admitted in < 4h, compared with those admitted in > 4h.

**Fig. 1:**
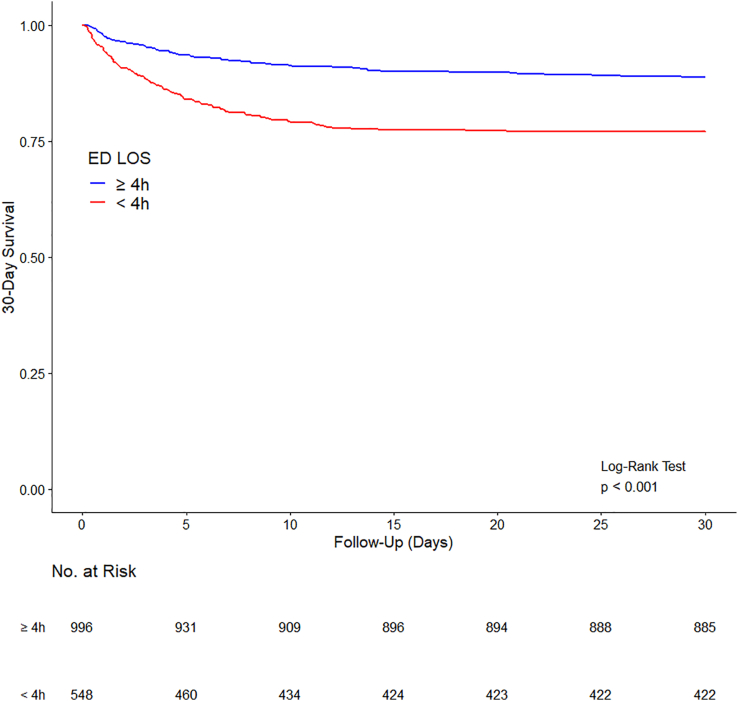
Kaplan Meier curve showing the difference in risk of death with time for patients admitted to the intensive care unit ＜4 h and ≧4 after arrival to the emergency department.

The increased risk of ICU, in-hospital, and 30-day mortality did not persist after adjusting for available confounders, particularly the adjusted mortality risk at 30 days (Table 3, Fig. 1).

**Fig. 2:**
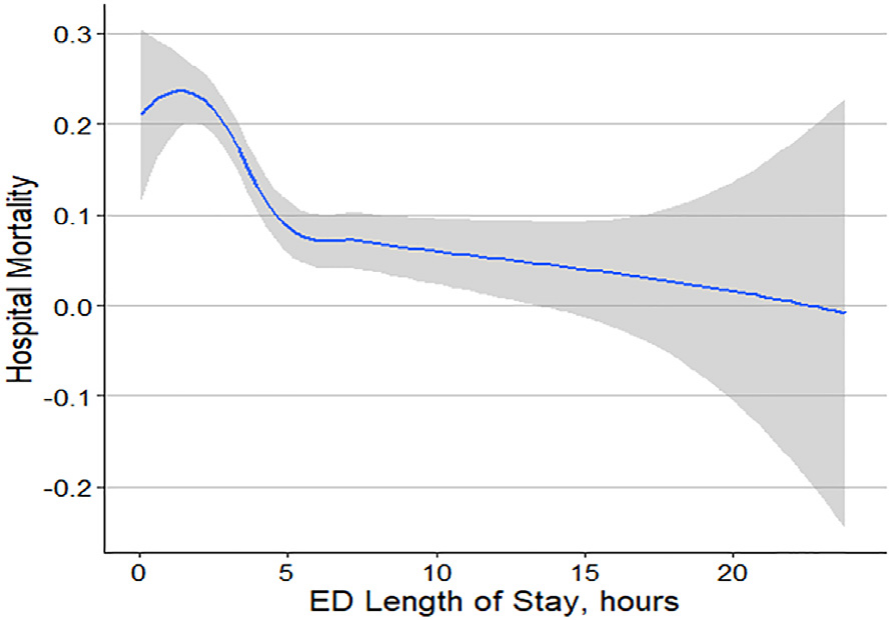
Figure showing non-liner relationship between risk of in-hospital mortality versus emergency department (ED) length of stay.

The association between in-hospital mortality and EDLOS was nonlinear (Fig. 2). Thus, the greatest risk of increased mortality was seen particularly with patients with an EDLOS of <2.5 h (Fig. 2).

## 4 Discussion

### 4.1 Summary of major findings

We carried out a retrospective observational study in a university affiliated hospital to examine the epidemiology of patients admitted directly to ICU in the era of NEAT.

We found that two thirds of the patients had an EDLOS >4 h, and this group of patients had less urgent ED triage categories, lower illness severity scores, and less use of mechanical ventilation in ICU. After adjustment for confounders, patients with an EDLOS>4 h had no difference in mortality but had an increased hospital length of stay compared to patients with an EDLOS of <4 h. The observed increased hospital length of stay is likely to be driven by the differences seen in nonsurvivors (Table 3).

Associations with EDLOS > 4 h included older age, the proportion of female gender, sepsis and respiratory diagnoses, and medical bed card admission.

### 4.2 Comparisons with previous studies

While the EDLOS in our study is more prolonged than reported by Crilly et al,^28^ the association of lower severity scores and use of mechanical ventilation is consistent with findings in a pre-NEAT era study.^14^ We did not find any association between prolonged EDLOS and increased mortality. This is consistent with pre-NEAT Australian data^14^ but contrasts with other international studies.^5^^,^^6^^,^^29^

Methodology may account for some differences including illness severity scoring systems and use of alternative models for risk adjustment. Alternatively, it may reflect differences in patient case mix or the presence of other unmeasured variables that we are not able to adjust for.

The group of patients with a prolonged EDLOS had significantly reduced illness severity compared with patients admitted promptly from the ED to ICU. It is notable that such disparity does not exist in other international studies and may contribute to the difference in observed results.^5^^,^^29^

### 4.3 Limitations

This was a single-centre retrospective study and as such a causal relationship cannot be attributed to the results. As noted, in other studies examining EDLOS for patients admitted to ICU,^14^ we could not account for clinician bias in selecting out patients appropriate for ICU referral and admission.

Illness severity scoring systems does not measure illness severity on arrival to ED or account for treatments and interventions occurring prior to ICU admission. However, we note that patients with EDLOS > 4h were triaged as being less unwell. The use of more formal illness severity scoring systems on arrival to ED may have produced different results. The time of referral to ICU was not captured in this study. This information may have provided additional insights into factors contributing to prolonged EDLOS.

We did not include deaths that occurred in the ED or patients who were admitted to the ward before ICU admission. Inclusion of these patient groups may have yielded different results.

The relatively small sample size conferred a reduction in the statistical power.

We are unable to comment on the use of vasopressors or renal replacement therapy in patients admitted to the ICU. This information was not routinely collected over the time period of the study.

Finally, the study period was before the COVID-19 pandemic and may not reflect the contemporary demands placed upon the ED-ICU interface.

### 4.4 Implications for clinical practice

We were unable to find an association between prolonged EDLOS and higher mortality and this may reflect shortfalls in the risk adjustment model.

Patients with higher illness severity were admitted more promptly to the ICU, suggesting a robust system of triage, referral and transfer to the ICU was in place for this group of patients.

Although we did not demonstrate an association between patients with prolonged EDLOS and higher mortality, this group represented the significant majority of the critically ill patients admitted to our ICU, approximately two thirds of the total cohort. Inherently, these patients will not be suitable for ward admission. Therefore, identifying factors that contribute to prolonged EDLOS will be of benefit in the setting of contemporary access block. Such factors might include delayed recognition of illness, clinical deterioration after ED triage, and access block to the ICU.

Given the preponderance of patients with sepsis among the group with a prolonged EDLOS and recent recommendations from the Australian Commission on Safety and Quality in Health Care,^30^ septic patients presenting to ED may be an appropriate group to target for early identification of need for ICU admission.

We found that approximately two thirds of all patients admitted to ICU present after 6 pm. A greater proportion of patients with prolonged EDLOS presented in winter months. These results may have implications for resource allocation and planning.

## 5 Conclusions

Approximately two thirds of ICU admissions have an ED length of stay of >4 h. This patient group were older and had a greater proportion of females, lower illness severity scores, and less urgent triage categories than patients admitted <4 h. EDLOS >4 h was not associated with increased mortality, but this patient group did have prolonged hospital length of stay.

## Credit author statement

**Jack D'Arcy:** Conceptualization,Writing - Original Draft; **Suzanne Doherty:** Writing - Review & Editing; **Luke Fletcher:** Investigation, Data Curation; **Ary Sepa Neto:** Methodology, Validation, Formal Analysis; **Daryl Jones:** Conceptualization, Supervision,Writing - Review & Editing.

## Conflicts of interest

The authors declare they have no conflict of interest.
